# Naghibione; A Novel Sesquiterpenoid with Antiplasmodial Effect from *Dorema hyrcanum *Koso-Pol. Root, a Plant Used in Traditional Medicine

**Published:** 2015

**Authors:** Farzaneh Naghibi, Saeedeh Ghafari, Somayeh Esmaeili, Kristina Jenett-Siems

**Affiliations:** a Traditional Medicine and Materia Medica Research Center (TMRC), Shahid Beheshti University of Medical Sciences, Tehran. Iran.; b School of Pharmacy, Shahid Beheshti University, of Medical Sciences, Tehran. Iran.; c Department of Traditional Pharmacy, School of Traditional Medicine, Shahid Beheshti University of Medical Sciences, Tehran. Iran.; d Institut Fuer Pharmazie (Pharmazeutische Biologie), Freie Universitaet Berlin, 14195. Berlin, Germany.

**Keywords:** Traditional medicine, *Dorema hyrcanum*, Antiplasmodial activity, Cytotoxicity, Sesquiterpenoid, Naghibione

## Abstract

Some *Dorema* species are used in Persian traditional medicine. In the present study the total extract from the roots of *Dorema hyrcanum *Koso-Pol. was investigated for its *in-vitro *(pLDH assay) and *in-vivo* (Peters’ 4-days suppressive test) antiplasmodial effects and assessed for cytotoxicity against the normal cell line MDBK (MTT test). The IC_50_ values for a chloroquine- sensitive (3D7) and a chloroquine- resistant (K1) strain of *Plasmodium falciparum* were 28.64 and 9.79 µg/mL, respectively. The inhibition percentage of the rodent parasite, *Plasmodium berghei,* on day 4 in mice was 77.9% and IC_50 _value on Madin–Darby bovine kidney cells (MDBK cells) was 59.84 µg/mL.

The total extract was subjected to a bioassay-guided fractionation protocol based on the *in-vivo* model which resulted in the isolation of an acetophenon (compound 1), one new sesquiterpenoid;

naghibione (compound 2) and two known sesquiterpenoid derivatives (compounds 3, 4). Their structures were elucidated by spectroscopic analysis, including 1D and 2D NMR experiments and ESI-MS. All compounds were evaluated for *in-vivo* antiplasmodial effect and the results revealed that naghibione showed good suppression activity, inhibiting 68.1 % of the parasite growth.

## Introduction

Malaria is a global public health problem whose main victims are children under five years of age in Africa. According to the latest WHO estimates, there were about 219 million cases of malaria in 2010. and an estimated 660000 deaths(1). 

Antimalarial drug resistance is a major concern for the global effort to control malaria. *P. falciparum* resistance to artemisinin has been detected in four countries in South East Asia: in Cambodia, Myanmar, Thailand and Vietnam. There is an urgent need to expand containment efforts in affected countries(1).

 Traditional medicine is a potential rich source of new drugs against malaria and other infectious diseases. It has made remarkable contributions to the development of potent antimalarial drugs (2).

Seven species of the *Dorema* (Apiaceae) are included in the flora of Iran(3). In Iran, *Dorema* species with the local names “*Oshagh”* or “*Vosha”*(4) are used in traditional medicine for different purposes such as pesticide, antihelminthic, expectorant and also for treatment of spleen and liver stiffness(5).

According to literature reviews, antimicrobial activity of *D. ammoniacum*(6) and the presence of weak antioxidant and antihemolytic activities in a hydroalcoholic extract of *D. aitchisonii* have been reported(7). *D. aucheri* is the first umbelliferous plant found to produce exudate flavonoids(8). Three sesquiterpene derivatives, one prenylated coumarin and two steroid glucosides were isolated from the aerial parts of *D. kopetdaghense*(9). There is a report about the phytochemical constituents of *D.hyrcanum* that mentions two glycosides, namely pleoside and hyrcanoside(10) and in an initial screening for anti-tumor agents by an *in-vitro* microbiological assay *D. hyrcanum *did not induce phage production(11). According to our studies there have been no scientific reports regarding antiplasmodial effect of these species.

In this search, for the first time, *D. hyrcanum *Koso-Pol., which is endemic to Iran, Turkmenistan and Afghanestan(12), has been investigated for its antiplasmodial effect by *in-vitro *and *in-vivo* assays and evaluated for its *in-vitro* cytotoxicity. A bio-assay guided isolation led to the identification of four compounds.

## Experimental

*General experimental procedures and materials*

NMR spectra were recorded on a Bruker DRX 500 spectrometer. Chemical shifts are given relative to TMS as an internal standard. ESI mass spectra were carried out on an Agilent 6210 ESI-TOF mass spectrometer (Agilent Technologies, Santa Clara, CA, USA). Semi preparative HPLC was performed on a Shimadzu instrument with PRC- ODS C18 (20 mm × 25 cm) column. Silica gel 60 (0.063- 0.2; 0.2- 0.5 mm; Merck) was used for column chromatography. All solvents for semi preparative HPLC were of technical grade and purified by distillation. 

*Plant material*

The roots of *D. hyrcanum* (Apiaceae), collected in June 2008 from Almed Valley in Golestan District, Iran, were identified by Mr. H. Moazeni from Traditional Medicine and Materia Medica Center (TMRC), Shahid Beheshti University of Medical Sciences, Iran. The voucher specimen 2495 (TMRC) of the plant has been deposited in the herbarium of the TMRC.

*Extraction*

The powdered dried root of *D. hyrcanum* was macerated in methanol (MeOH), for 24 hours with constant shaking, at room temperature. The filtrates of total extract were evaporated to dryness and investigated for its *in-vitro* and *in-vivo* antiplasmodial and cytotoxic effects.

*Biological assays*

*In-vitro antiplasmodial activity*

Antiplasmodial activity of the total extract was determined against the chloroquine-resistant (K1) and chloroquine-sensitive (3D7) strains of *Plasmodium falciparum* that were continuously cultured according to the methods described by Trager and Jensen (13). Plant extract was assessed for antiplasmodial activity *in-vitro* in human blood using parasite lactate dehydrogenase method (pLDH) with slight modifications(14, 15). The range of examined concentration was from 64 µg/mL to 125 ng/mL. A solution of chloroquine diphosphate and artemisinin served as positive control. The test was performed in duplicate. Absorbance was measured with an ELISA plate reader at 630 nm. The percentage inhibition at each concentration was determined and the mean of IC_50_ value of parasite viability was calculated using Probit analysis(16). 

*In-vivo antimalarial assay*

Peters’ 4-day suppressive test against NICD strain of* Plasmodium berghei* infection in mice was employed(17) for evaluating of the samples of *D.hyrcanum* antiplasmodial activities. Adult male albino mice from the Pasteur Institute of Iran were housed under standard environmental conditions and fed with standard pellets and water. All the procedure was accepted by Shahid Beheshti University of Medical Sciences Ethics Committee and in accordance with the principles for laboratory animal use and care in the European Community guidelines. 

Briefly, the parasites (blood contained parasites) were maintained by serial passage of blood from mouse to mouse. Adult male albino mice weighing 20–25 g were inoculated by intra-peritoneal (I.P) injection with 1×10^7^ infected erythrocytes. The mice were randomly divided into groups of five per cage and treated during consecutive days with 10 mg/mL of the sample by I.P injection for 4 days. Two control groups were used in this experiment, one treated with chloroquine at dose of 20 mg/Kg as a positive control while the other group was kept untreated as a negative group. On day 5 of the test, thin blood smears were prepared and blood films were fixed with methanol. The blood films were stained with Giemsa, and then microscopically examined. Percentage of parasitaemia was counted based on infected erythrocytes calculated per 1000 erythrocytes.

The mice were inoculated by intra-peritoneal (I.P) injection with 10^7^ infected erythrocytes. The mice were treated during consecutive days with 10 mg/Kg of the sample by I.P injection for 4 days. Two control groups were used in this experiment, one treated with chloroquine at dose of 20 mg/Kg as a positive control while the another group was kept untreated as a negative group. Percentage of parasitaemia was counted based on infected erythrocytes calculated per 1000 erythrocytes and the inhibition percentage of each group expressed in relation to the untreated group.

*In-vitro cytotoxicity assay*

Cytotoxicity of samples was determined using MDBK cells(2, 18) by the colorimetric methyl thiazole tetrazolium (MTT) assay (19, 20) and scored as a percentage of absorbance reduction at 570 nm of treated cultures versus untreated control cultures. Tests were run in triplicate and Tamoxifen was used as a positive control. IC_50_ values were calculated from the drug concentration–response curves. 

*Bioassay guided fractionation and isolation*

*D. hyrcanum* was subjected to a bioassay-guided fractionation protocol based on the *in-vivo* model. For this purpose the roots of *D. hyrcanum* (750 g) were macerated in ethyl acetate (EtOAc) for 24 hours at room temperature with constant shaking (2: 1) for 3 times(21). After filtration, the extract was concentrated to yield 180g of a brown residue. Compound 1 (50 mg) precipitated when the ethyl acetate extract was evaporated. The ethyl acetate extract was fractionated twice with 500 mL water. The dried ethyl acetate fraction was separated by silica gel column chromatography (Silica gel 60, 0.2-0.5 mm; Merck) eluting with hexane, hexane- dichloromethane (DCM) (5: 5), DCM, DCM- EtOAc (5:5), EtOAc, EtOAc- MeOH (5:5), MeOH to give five fractions. The ethyl acetate extract, as well as fractions 2 (1.20 g) and 3 (40 g), 4 (3 g) which were eluted with hexane- DCM (5:5), DCM and EtOAc- MeOH (5:5) respectively, were evaluated for *in-vivo* antiplasmodial and cytotoxicity activity. 

Fraction 2 was separated using silica gel column chromatography (0.063- 0.2 mm; Merck) eluting with hexane, a gradient of hexane- chloroform (9:10, 8:2, 6:4, 40:60, 1:9 to pure chloroform), a gradient of chloroform- EtOAc (9:1, 8:2 to pure EtOAc), EtOAc- MeOH (5:5) and MeOH to give 14 fractions. Compound 2 (200 mg) was isolated from fraction 2- 6 (237 mg) which was eluted with hexane- chloroform (1:9) to 100% chloroform.

Fraction 3 was separated by silica gel column chromatography (0.02- 0.5 mm; Merck) eluting with a gradient of petroleum benzene- DCM (3:7, to pure DCM), a gradient of DCM- EtOAc (9:1, 8:2, 7:3, 6:4, 5:5, 3:7 to pure EtOAc), EtOAc- MeOH (5:5) and MeOH to give 8 fractions. Fraction 3-2 (2 g), which was eluted with DCM- EtOAc (9:1), was separated by silica gel column chromatography (0.02- 0.5 mm; Merck) eluting with a gradient of petroleum benzene- DCM (8:2, 7:3, 6:4, 5:5, 4:6, 3:7 to pure dichloromethane), gradients of dichloromethane- EtOAc (9:1, 8:2, 7:3, 6:4, 5:5 to pure EtOAc), EtOAc- MeOH (5:5) and MeOH. There were obtained 7 sub fractions. Fraction 3-2-3 (230 mg) was separated by semi preparative HPLC [MeOH 100%, 30 min, flow rate 8 mL/min] and then isolated the subfraction with R_t_ = 23.5min (76 mg). It was purified on a silica gel column (0.63- 0.2 mm; Merck) wi'th a gradient of petroleum benzene- DCM (7:3, 4:6 to pure dichloromethane), a gradi'ent of dichloromethane- EtOAc (8:2, 7:3, 6:4, 5:5 to pure EtOAc), EtOAc- MeOH (5:5), MeOH to yield compounds **3 **(18 mg) and **4 **(20 mg). All pure compounds were evaluated for *in-vivo* antiplasmodial effects. 

4-methoxy-6-hydroxyacetophenone-2-O-β-D-gentiobioside (1): Yellow crystal, ^1^H-NMR (500 MHz, CDCl_3_): δ ppm= 6.14 (s, H-3), 6.25 (s, H-5), 5.19 (d, *J*= 8.5 Hz, H-1'), ^13^C-NMR: δ =206.4 (C-7), 166.8 (C-4), 164.5 (C-2), 160.2 (C-6), 106.9 (C-1), 103.2 (C-1''), 100.0 (C-1'), 96.0 (C-5), 95.0 (C-3), 76.4 (C-3'), 76.1 (C-5''), 76.0 (C-5,'C-3''), 74.0 (C-2''), 73.2 (C-2'), 70.1 (C-4''), 69.6 (C-4'), 68.8 (C-6'), 61.2 (C-6''), 56.3 (C-OMe), 33 (C-8); HR‑ESI‑TOF‑MS (positive): m/z = 506.1631 [M + Na]^+^ (calcd. For C_21_H_30_O_14_ Na: 506.4533).

1(2-hydroxy-4-methoxy)- 3,7,11- trimethyl-3-vinyl-6*(E)*, 10 dodecadiene- 1- dione (2): Yellow oil; ^1^H- (500 MHz, CDCl_3_) and ^13^C‑NMR (125 MHz) data: see Table 2; HR‑ESI‑TOF‑MS (positive): m/z = 393.2438 [M + Na]^+^ (calcd. For C_24_H_34_O_3 _Na: 393.5134). 

2,3-dihydro-7-methoxy-2S*,3R*-dimethyl-2-[4,8-dimethyl-3(*E*),7-nonadienyl]-furo[3,2-*c*]coumarin (3): yellow oil, ^1^H-NMR (500 MHz, CDCl_3_): δ ppm = 7.52 (d, *J* = 8.5 Hz, H-9), 6.85 (dd,* J* = 1.5,8.5 Hz, H-6), 6.83(d,* J* =1.5, H-8), 5.08 (t, overlap, H-3'), 5.05 (overlap, H-7'), 3.86 (s, H-OMe), 3.27 (q, *J* =7.0 Hz, H-3), 2.09 (m, H-2'), 2.01 (m, H-6'), 1.94 (m, H-5'), 1.67 (s, H-9'), 1.59 (s, H-4' Me, H- 8' Me), 1.77 (m, H-1'), 1.44 (s, H-2Me),^ 13^C-NMR: δ = 165.4 (C- 9b), 165.2 (C4), 163.2 (C-7), 156.9 (C- 5a), 131.9 (C-8'), 136.0 (C-4'), 124.2 (C-7'), 122.8 (C-3'), 123.7 (C-9), 112.1 (C- 8), 106.3 (C-9a), 103.7 (C-3a), 100.7 (C-6), 96.6 (C-2), 55.2 (C-OMe), 39.0 (C-5'), 42.0 ( C-3), 41.5 (C-1'), 25.7 (C-9'), 22.1 (C-2 '), 20.2 (C-2Me), 17.7 (C-8'Me), 16.0 (C-4'Me), 13.7; HR‑ESI‑TOF‑MS (positive): m/z = 419.2221 [M + Na]^+^ (calcd. For C_25_H_32_O_4 _Na: 419.5077).

2,3-dihydro-7-methoxy-2R*,3R*dimethyl-2-[4,8-dimethyl-3(*E*),7-nonadienyl]-furo[3,2-*c*]coumarin (4): yellow oil, ^1^H-NMR (500 MHz, CDCl_3_): δ ppm= 7.52 (d, *J* = 8.5 Hz, H-9), 6.85 (dd,* J* = 1.5Hz, H-6), 6.83 (dd,* J* =1.5,8.5, H-8), 5.19 (t, overlap, H-3'), 5.05 (overlap, H-7'), 3.86 (s, H-OMe), 3.22 (q, *J* =7.0 Hz, H-3), 2.24 (m, H-2'), 2.01 (m, H-6'), 1.94 (m, H-5'),1.67 (s, H-9'), 1.63 (s, H-4' Me), 1.60 (s, H- 8' Me), 1.90 (m, H-1'), 1.48(s, H-2Me),^ 13^C-NMR: δ = 165.4 (C- 9b), 165.2 (C4), 163.2 (C-7), 156.9 (C- 5a), 131.9 (C-8'), 136.0 (C-4'), 124.2 (C-7'), 122.8 (C-3'), 123.7 (C-9), 112.1 (C- 8), 106.3 (C-9a), 103.7 (C-3a), 100.7 (C-6), 96.6 (C-2), 55.2 (C-OMe), 39.0 (C-5'), 44.4 ( C-3), 35.4 (C-1'), 25.7 (C-9'), 23.1 (C-2'), 25.7 (C-2Me), 17.7 (C-8'Me), 16.0 (C-4'Me), 13.7; HR‑ESI‑TOF‑MS (positive): m/z = 419.2221 [M + Na]^+^ (calcd. For C_25_H_32_O_4 _Na: 419.5077).

## Results and Discussion

In this study for the first time *D. hyrcanum *was evaluated for its antiplasmodial activity. In a screening program, its methanolic extract was tested against a chloroquine- sensitive strain of *Plasmodium falciparum* (3D7) as well as a chloroquine- resistant strain (K1) and showed IC_50_ values of 28.64 and 9.79 µg/mL, respectively. Furthermore it was evaluated by Peters’ 4-day suppressive test and revealed 77.9% inhibition of *P. berghei* on day 4 in mice at a concentration of 10 mg/Kg. In a cytotoxicity assay against MDBK cells, it showed an IC_50 _value of 59.84 µg/mL. 

A bioassay-guided fractionation protocol based on the *in-vivo* model (Peters’ test) was assessed in order to isolate the active compounds. Therefore, the ethyl acetate extract, some of its selected fractions and pure compounds were evaluated for antiplasmodial activity by the *in-vivo* test and for cytotoxicity (Table 1). 

Fractions 2, 3 and 4 were exhibited higher antiplasmodial activity and lower cytotoxicity than that of the total extract. All pure compounds were evaluated for *in-vivo* antiplasmodial effect and the results revealed that compound 2 showed good suppression activity, inhibiting 68.1 % of the parasite growth.

**Table 1:** *In* *-* *vivo* antiplasmodial activity and cytotoxic effects of ethyl acetate extract, selected fractions of* D. hyrcanum* and pure compounds

**Sample**	**Antiplasmodial activity** ***Plasmodium berghei*** **% inhibition± SD**	**Cytotoxic effect** **MDBK Cell** **IC**_50_ ** (µg/mL)** ** ±** ** SD**
Ethyl acetate extract	73(±0.5)	69.67
Fraction 2	61(±1.2)	>400
Fraction 3	51.7 (±2)	104.82
Fraction 4	35.2 (±3.9)	188.04
Compound 1	10.1(± 9.6)	
Compound 2	61.8 (± 2.5)	>100
Compound 3	29.3 (±0.5)	
Compound 4	23.3 (±0.5)	
Chloroquine	100 (±4.6)	
Tamoxifen		4.76

* Not done

The bio-assay guided fractionation led to the isolation of compound 2**, **which was obtained from fraction 2 as yellow oil. It displayed a quasi-molecular ion peak of *m/z* = 393.2438 [M+Na]^ +^ in its HR-ESI-TOF MS, corresponding to the formula C_24_H_34_O_3_. The ^1 ^H and ^13 ^C NMR resonances (Table 2) of compound 2 were assigned by different 2D NMR experiments. The ^1^H NMR spectrum of 2 showed resonances characteristic for four methyl singlets at δ 1.16, 1.58, 1.59, and 1.68, a methoxy singlet at δ 3.83, and four olefinic resonances at 5.09 (overlapped), 4.95 (1H, d, *J=* 17.5), 5.00 (1H, d,* J=*11.0 Hz), 5.88 (1H, dd, *J=*11.0 and 17.5 Hz). Three aromatic protons at δ 6.40 (br s), 6.42 (1H, dd, *J= *9.5, 2.3 Hz) and 7.65 (1H, d, *J= *9.5 Hz) suggested the presence of a 1, 2, 4-trisubstituted benzene ring, which was supported by the ^13^C NMR spectrum. The side chain of this ring was also assigned through ^13^C NMR, HMQC, ^1^H-^1^H COSY, and HMBC experiments. In the HMBC spectrum of 2, the correlations of H-2 (*δ*_H_ 2.87) with C-1 (*δ *C 204.2), C-3 (40.1), and C-13 (145.8); H-8 (*δ*_H_ 1.95) with C-7 (*δc* 135.5) and C-9 (*δc* 26.5); H-13 (*δ*_H_ 5.88) and H-14 (*δ*_H_ 4.97) with C-3 (40.1); OH (*δ*_H_ 13.1) with C-3' (*δ*_C _100.5), C-1' (*δ*_C_ 115.0), and C-2' (*δ*_C_ 166.2); and H-6' (*δ*_H_ 7.65) with C-1 (*δ*_C_ 204.2) and C-4' (*δ*_C_ 165.85) confirmed the structure of compound 2 (Figure 2). 

Therefore, the structure of compound 2 was elucidated as 1-(2-hydroxy-4-methoxyphenyl)-3, 7, 11-trimethyl- 3- vinyl- 6(*E*), 10- dodecadiene-1- dione and named naghibione.

A congener with a 4-hydroxy group instead of the methoxy group was obtained from *Ferula ferulaeoides* (22) and with a carbonyl group on C9 from *Dorema kopetdaghense* (9) previously. In the best our knowledge the same compounds have not been tested *in-vivo* and *in-vitro* for potential antiplasmodial activities. 

Among the reviewed compounds exhibited high activity (IC_50_≤ 2µM), sesquiterpenes should be considered as lead compounds for further investigation(23, 24).

Compounds 1, 3 and 4 were known compounds, and their structures were elucidated as 4-methoxy-6-hydroxyacetophenone-2-O-β-D-gentiobioside; 2,3-dihydro-7-methoxy-2S*,3R*-dimethyl-2-[4,8-dimethyl-3(*E*),7-nonadienyl]-furo[3,2-*c*]coumarin and 2,3-dihydro-7-methoxy-2R*,3R*dimethyl-2-[4,8-dimethyl-3(*E*),7-nonadienyl]-furo[3,2-*c*]coumarin, respectively, by comparison with literature data (10, 25). 

**Table 2:** ^1 ^H NMR and ^13 ^C NMR Data of Compound 2 (CDCl_3_, 500 MHz)^a^*.*

**Position**	***δ***_H_	***δ***_C_
1	-	204.2
2	2.87 (d, 14.0 Hz), 2.90 (d, 14.0 Hz)	47.03
3	-	40.1
4	1.53 b	40.8
5	1.95 b	23.3
6	5.09 b	124.5
7	-	135.5
8	1.95 b	39.4
9	2.05 b	26.5
10	5.09 b	124.5
11	-	131.9
12	1.68 (s)	25.2
13	5.88 (dd, 11.0; 17.5 Hz)	145.8
14	4.95 (d, 17.5 Hz), 5.00 (d, 11.0 Hz)	112.2
1'	-	115.0
2'	-	166.2
3'	6.40 (br s)	100.5
4'	-	165.9
5'	6.42 (dd, 9.5; 2.3 Hz)	107.4
6'	7.65 (d, 9.5Hz)	131.3
3Me	1.16 (s)	23.3
7Me	1.58 (s)	17.7
11Me	1.59 (s)	17.6
OCH_3_	3.83 (s)	55.54

a *J*-values are in parentheses and reported in Hz; chemical shifts are given in ppm; assignments were confirmed by HMQC, HMBC experiments.

b overlapped with other signals.

**Figure 1:**
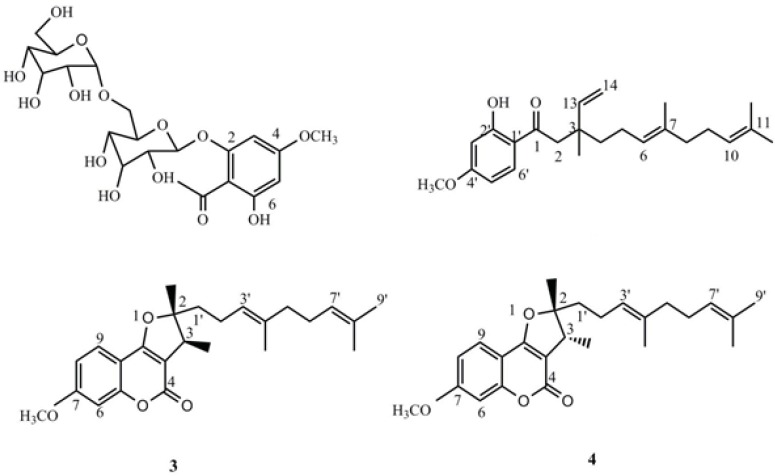
Structures of compounds 1-4

**Figure 2:**
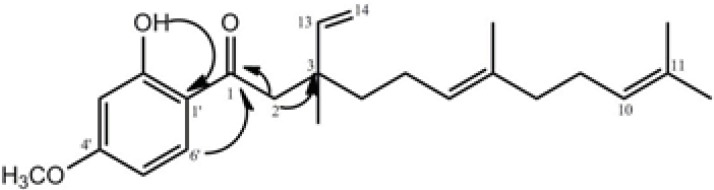
Selected HMBC correlations for compound 2

## Conclusion

In this study for the first time, *Dorema hyrcanum *Koso-Pol. was evaluated for its antiplasmodial activity and naghibione (compound 2); a new sesquiterpenoid with antiplasmodial activity has been isolated. Furthermore, two known sesquiterpenoids 3 and 4 were for the first time isolated from this genus. 

Further studies in order to evaluate the mode of action of the pure compounds are under way. 
